# Structure of the type V-C CRISPR-Cas effector enzyme

**DOI:** 10.1016/j.molcel.2022.03.006

**Published:** 2022-05-19

**Authors:** Nina Kurihara, Ryoya Nakagawa, Hisato Hirano, Sae Okazaki, Atsuhiro Tomita, Kan Kobayashi, Tsukasa Kusakizako, Tomohiro Nishizawa, Keitaro Yamashita, David A. Scott, Hiroshi Nishimasu, Osamu Nureki

**Affiliations:** 1 Department of Biological Sciences, Graduate School of Science, The University of Tokyo, 7-3-1 Hongo, Bunkyo-ku, Tokyo 113-0033, Japan; 2 Structural Biology Division, Research Center for Advanced Science and Technology, The University of Tokyo, 4-6-1 Komaba, Meguro-ku, Tokyo 153-8904, Japan; 3 PeptiDream Inc., 3-25-23 Tonomachi, Kawasaki-ku, Kawasaki City, Kanagawa Prefecture 210-0821, Japan; 4 Graduate School of Medical Life Science, Yokohama City University, Yokohama 230-0045, Japan; 5 MRC Laboratory of Molecular Biology, Francis Crick Avenue, Cambridge CB2 0QH, UK; 6 Arbor Biotechnologies, Cambridge, MA 02139, USA; 7 Inamori Research Institute for Science, 620 Suiginya-cho, Shimogyo-ku, Kyoto 600-8411, Japan

**Keywords:** CRISPR, Cas12c, cryo-EM

## Abstract

RNA-guided CRISPR-Cas nucleases are widely used as versatile genome-engineering tools. Recent studies identified functionally divergent type V Cas12 family enzymes. Among them, Cas12c2 binds a CRISPR RNA (crRNA) and a *trans*-activating crRNA (tracrRNA) and recognizes double-stranded DNA targets with a short TN PAM. Here, we report the cryo-electron microscopy structures of the Cas12c2–guide RNA binary complex and the Cas12c2–guide RNA–target DNA ternary complex. The structures revealed that the crRNA and tracrRNA form an unexpected X-junction architecture, and that Cas12c2 recognizes a single T nucleotide in the PAM through specific hydrogen-bonding interactions with two arginine residues. Furthermore, our biochemical analyses indicated that Cas12c2 processes its precursor crRNA to a mature crRNA using the RuvC catalytic site through a unique mechanism. Collectively, our findings improve the mechanistic understanding of diverse type V CRISPR-Cas effectors.

## Highlights

• Cryo-EM structures of the Cas12c2–guide RNA and Cas12c2–guide RNA–target DNA complexes
• The crRNA and tracrRNA of Cas12c2 form an unexpected X-junction structure
• Cas12c2 uses two arginine residues to recognize a single T nucleotide in the PAM
• Cas12c2 processes its pre-crRNA at the RuvC active site via a unique mechanism

## Graphical abstract

**Figure:**
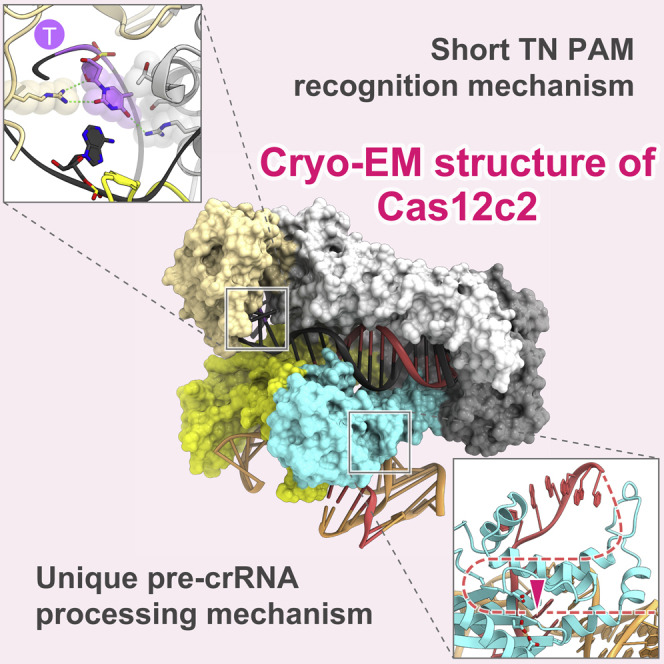

## Teaser

Kurihara et al. report the cryo-EM structures of the Cas12c2–guide RNA complex and the Cas12c2–guide RNA–target DNA complex, providing mechanistic insights into the TN PAM recognition by Cas12c2. Furthermore, biochemical analysis revealed that Cas12c2 processes its pre-crRNA through a unique mechanism.

## Introduction

The CRISPR-Cas systems in bacteria and archaea provide adaptive immunity against foreign nucleic acids and are divided into two classes (classes 1 and 2) and six types (types I–VI) (Hille et al., 2018; Makarova et al., 2020). The class 2 systems include types II, V, and VI, in which Cas9, Cas12, and Cas13 function as effector enzymes responsible for the interference, respectively. Cas9 associates with dual-guide RNAs (CRISPR RNA [crRNA] and *trans*-activating crRNA [tracrRNA]) or a synthetic single-guide RNA (sgRNA) and cleaves double-stranded DNA (dsDNA) targets with a sequence complementarity to a 20-nucleotide segment in the guide RNA (Gasiunas et al., 2012; Jinek et al., 2012). Cas9 exhibits robust DNA cleavage activity in eukaryotic cells and is widely used as a powerful genome-editing tool (Cong et al., 2013). However, the targetable genomic regions are limited, since Cas9 requires an NGG (N is any nucleotide) protospacer-adjacent motif (PAM) for target dsDNA recognition.

Recent studies identified functionally divergent type V Cas12 effector proteins (Cas12a–Cas12k) (Zetsche et al., 2015; Shmakov et al., 2015; Yan et al., 2019; Strecker et al., 2019; Makarova et al., 2020; Pausch et al., 2020). While the Cas12 proteins commonly have a single RuvC nuclease domain, they share a low sequence similarity, except for the RuvC domain, and exhibit diverse biochemical activities. Cas12a (also known as Cpf1) cleaves dsDNA targets with a TTTV (V is A, G, or C) PAM and is used for genome-editing applications (Zetsche et al., 2015). The Cas12c (also known as C2c3) proteins, such as Cas12c1, Cas12c2, and OspCas12c, exhibit RNA-guided dsDNA interference activity (Shmakov et al., 2015; Yan et al., 2019). Notably, unlike the other Cas12 enzymes, Cas12c2 and Cas12c1/OspCas12c recognize short TN and TG PAMs, respectively, thereby potentially expanding the target space in genome editing. The Cas12 enzymes employ guide RNAs with distinct architectures. Cas12a, Cas12i, and Cas12j (CasΦ) use a crRNA, whereas Cas12b (C2c1), Cas12c, Cas12e (CasX), and Cas12f (Cas14) use dual-guide RNAs (crRNA and tracrRNA).

In the CRISPR-Cas enzymes, crRNAs are transcribed as precursor crRNAs (pre-crRNAs), which are processed to mature crRNAs through diverse mechanisms. In the type II system, the Cas9-bound crRNA-tracrRNA duplexes are processed by the host RNase III nuclease (Deltcheva et al., 2011). Unlike Cas9, most Cas12 enzymes, such as Cas12a, Cas12i, Cas12j, and Cas12c, can process their own pre-crRNAs without the host RNase III. Cas12a and Cas12i process the 5′ end of their pre-crRNAs at their Wedge (WED) domain, through metal-independent, acid-base catalytic mechanisms (Swarts et al., 2017; Zhang et al., 2020; Huang et al., 2020; Zhang et al., 2021), whereas Cas12j processes the 5′ end of its pre-crRNA at the RuvC domain through a metal-dependent mechanism (Pausch et al., 2020). A recent study reported that Cas12c processes the 3′ end of its pre-crRNA through a unique ruler mechanism (Harrington et al., 2020). Unlike Cas9, Cas12c requires the tracrRNA (also known as a short-complementarity untranslated RNA [scoutRNA]), but not the host RNase III, for the pre-crRNA processing (Harrington et al., 2020). However, the mechanisms of the PAM recognition and pre-crRNA processing by Cas12c remain unknown due to the lack of structural information of the Cas12c family enzymes.

Here, we present the cryo-electron microscopy (cryo-EM) structures of the Cas12c2–guide RNA binary complex and the Cas12c2–guide RNA–target DNA ternary complex, at overall resolutions of 3.0 Å and 2.7 Å, respectively. The structures provide mechanistic insights into the PAM recognition and pre-crRNA processing by Cas12c2. Comparisons with the other Cas12 enzymes highlight the structural and functional diversity of type V CRISPR-Cas enzymes. Furthermore, our findings will contribute to broadening the target range in genome-engineering technologies.

## Results

### Biochemical characterization of Cas12c2

Previous studies reported that, while the Cas12c proteins (Cas12c1, Cas12c2, and OspCas12c) mediate dsDNA interference in bacterial cells (Yan et al., 2019), they lack *in vitro* dsDNA cleavage activity (Harrington et al., 2020). To biochemically characterize Cas12c, we performed *in vitro* DNA cleavage assays, using the purified Cas12c proteins (Cas12c1, Cas12c2, and OspCas12c), their guide RNAs (crRNA and tracrRNA), and circular and linearized plasmid DNAs containing an 18-nucleotide target sequence with the TG PAM. Consistent with a previous study (Harrington et al., 2020), Cas12c2 and OspCas12c cleaved neither circular nor linear DNA targets (Figure S1A), indicating that Cas12c2 and OspCas12c induce neither nicking nor dsDNA cleavage. In contrast, Cas12c1 cleaved both DNA targets (Figure S1A), indicating that it cleaves the target strand (TS) and the non-target strand (NTS) in the dsDNA target. Among the Cas12c proteins, we selected Cas12c2 for further biochemical and structural studies, since Cas12c2 recognizes the short TN PAM. To examine whether Cas12c2 binds to the guide RNA and a dsDNA target, we mixed the purified Cas12c2 with the sgRNA, in which a 35-nucleotide crRNA and a 71-nucleotide tracrRNA are fused with a GAAA tetraloop, and a 33-bp dsDNA containing a 17-nucleotide target sequence with the TG PAM, and then analyzed the mixture by size-exclusion chromatography. The Cas12c2–sgRNA–target DNA complex was eluted from the column as a single peak, indicating that Cas12c2 and the sgRNA form an effector complex that recognizes its dsDNA target (Figure S1B). These results confirmed that the Cas12c2–sgRNA complex binds, but does not cleave, its dsDNA targets, at least under the tested conditions.

### Cryo-EM structure of the Cas12c2–guide RNA–target DNA ternary complex

**Figure 1:**
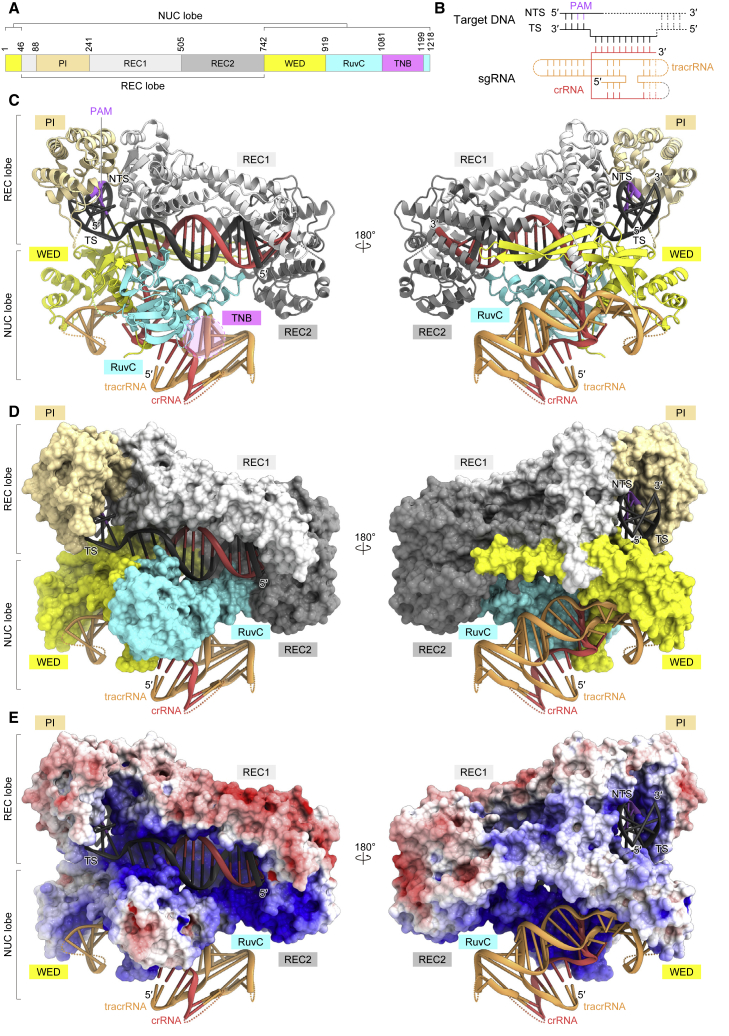
Cryo-EM structure of the Cas12c2–guide RNA–target DNA complex (A) Domain structure of Cas12c2. (B) Schematic of the guide RNA and target DNA. The disordered regions are indicated by dashed lines. TS, target strand; NTS, non-target strand. (C) Overall structure of the Cas12c2–sgRNA–target DNA complex. The disordered regions are indicated by dotted lines. The predicted location of the TNB domain is indicated by a magenta circle. (D and E) Molecular surface (D) and electrostatic surface potential (E) of the Cas12c2–sgRNA–target DNA complex. See also Figures S1–S3.

To elucidate the molecular mechanism of Cas12c2, we determined the cryo-EM structure of Cas12c2 in complex with a 110-nucleotide sgRNA and a 33-bp dsDNA target with a TG PAM, at an overall resolution of 2.7 Å (Figures 1A–1E and S2A–S2E; Table 1). Cas12c2 adopts a bilobed architecture consisting of recognition (REC) and nuclease (NUC) lobes. The REC lobe has the REC1, REC2, and PAM-interacting (PI) domains, and the NUC lobe comprises the WED and RuvC domains. Residues 1081–1198, which are inserted within the RuvC domain and correspond to the target nucleic acid-binding (TNB) domain of the other Cas12 enzymes, are disordered in the present structure (Figures S3A and S3B). The sgRNA–target DNA heteroduplex is accommodated within the positively charged central channel formed by the REC1, REC2, and RuvC domains (Figures 1C–1E). The PAM-containing DNA duplex (the PAM duplex) is surrounded by the WED, REC1, and PI domains. The sgRNA scaffold binds to a positively charged surface formed by the WED and RuvC domains (Figures 1C–1E).

**Table 1:** Data collection, processing, model refinement, and validation

**Data collection and processing**		

Sample	Cas12c2–sgRNA–DNA	Cas12c2–sgRNA
EMDB ID	EMD-31808	EMD-31807
PDB ID	7V94	7V93
Microscope	Titan Krios G3i	
Detector	Gatan K3 camera	
Magnification	105,000	
Voltage (kV)	300	
Electron exposure (e^–^/Å^2^)	50	65
Defocus range (μm)	−0.8 to −1.6	
Pixel size (Å)	0.83	
Symmetry imposed	*C*1	
Initial particle images (no.)	1,465,975	2,852,137
Final particle images (no.)	534,196	289,394
Map resolution (Å)	2.7	3.0
FSC threshold	0.143	
Map sharpening *B* factor (Å^2^)	−77.5	−108.1

**Model building and refinement**		

Model composition		
Protein atoms	8,440	7,260
Nucleic acid atoms	2,399	1,532
Rmsds		
Bond lengths (Å)	0.0083	0.0100
Bond angles (°)	1.46	1.41

**Validation**		

MolProbity score	2.15	2.35
Clashscore	5.80	7.42
Rotamer outliers (%)	9.51	14.08
Ramachandran plot		
Favored (%)	97.56	97.7
Allowed (%)	2.44	2.19
Outliers (%)	0.00	0.11

Inside the complex molecule, the amino-acid residues of Cas12c2 and the nucleotides of the sgRNA and the target DNA are clearly visible in the density map at high local resolutions (up to ∼2.5 Å) (Figure S2D). In contrast, we observed relatively poor densities for the peripheral regions of the complex molecule, such as the PI domain (residues 88–240) and the sgRNA scaffold (nucleotides [−41]–[−33] and [−27]–[−14]), indicating the flexibility of these regions (Figure S2D). We did not observe clear densities for residues 1–4, 141–144, 558–566, 913–918, 1081–1198, and 1218 of Cas12c2; nucleotides (−93), (−69)–(−67), (−41)–(−33), and (−27)–(−14) of the sgRNA; nucleotides (−8)–(−1), and 24–25 of the TS; and (−8^∗^)–(−7^∗^) and 3^∗^–25^∗^ of the NTS, which were not included in the final model.

### Domain structures

The REC1 domain comprises 11 α helices and two β strands (Figure S3A). The PI domain contains eight α helices and is inserted between the two β strands in the REC1 domain (Figure S3A). The REC2 domain comprises 10 α helices (Figure S3A). A Dali search (Holm and Rosenström, 2010) revealed that the REC1, REC2, and PI domains of Cas12c2 lack structural similarity with any other known proteins, including those of other Cas12 enzymes. Cas12b and Cas12e lack the PI domain and instead use the REC and WED domains for the PAM recognition (Yang et al., 2016; Liu et al., 2019). Although Cas12a has the PI domain, it is structurally unrelated to that of Cas12c2, and is inserted within the WED domain, rather than the REC1 domain (Swarts and Jinek, 2019) (Figure S3A).

The WED domain adopts an oligonucleotide/oligosaccharide-binding (OB) fold, consisting of seven β strands flanked by an α helix, with an additional five α helices and a β strand (Figure S3A). The additional β strand interacts with the core β strands (β3 and β5) to form a β-barrel structure. The strands β2, β6, and β7 of the Cas12c2 WED domain are longer than those of the other Cas12 WED domains and interact with the REC2 domain. Cas12a has a β-hairpin-like loop between the strands β6 and β7, which is responsible for the pre-crRNA processing (Figure S3A). The WED domain of Cas12c2 has an additional α-helical subdomain, which is inserted between the strands β3 and β4 and interacts with the tracrRNA scaffold.

The RuvC domain contains an RNase H fold, consisting of a conserved five-stranded mixed β sheet flanked by four α helices and an additional α helix and two β strands (Figure S3A). The conserved catalytic residues (Asp928, Glu1014, and Asp1201) are located at positions similar to those of the other Cas12 enzymes (Yamano et al., 2016; Yang et al., 2016; Stella et al., 2017; Swarts and Jinek, 2019). A linker region (residues 964–983, referred to as the α1–α2 linker) between the helices α1 and α2 interacts with the REC2 domain and the guide RNA–target DNA heteroduplex.

Cas12 enzymes commonly contain the TNB domain (also known as the Nuc or target-strand loading [TSL] domain) inserted between the strand β5 and the helix α4 in the RuvC core fold (Figures S3A and S3B). The TNB domains adopt distinct protein folds and contribute to the target DNA loading into the RuvC active site (Yang et al., 2016; Swarts et al., 2017; Liu et al., 2019; Swarts and Jinek, 2019). A sequence comparison of the Cas12c proteins revealed the presence of putative zinc-finger motifs (CXXC and CXXXXC) between the strand β5 and the helix α4 in the Cas12c2 RuvC domain, as also observed in Cas12e (Liu et al., 2019) and Cas12f (Takeda et al., 2021) (Figure S3B). An X-ray fluorescence elemental analysis of the purified Cas12c2 protein revealed that Cas12c2 contains zinc ions (Figure S3C). These results suggested that Cas12c2 has the TNB domain with the CCCC-type zinc finger, in which a zinc ion is coordinated by the four cysteine residues (C1089, C1092, C1189, and C1194), although the TNB domain is not resolved in the density map, probably due to its flexibility.

### RNA scaffold architecture

**Figure 2:**
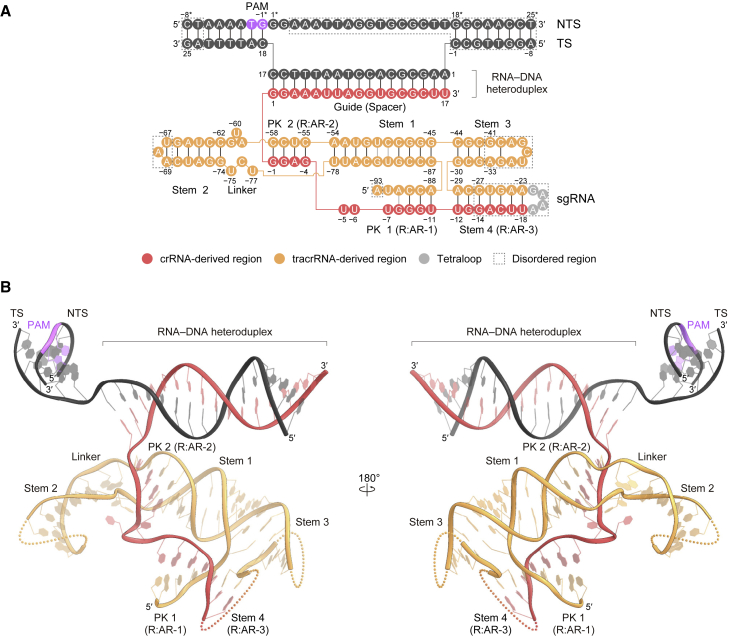
Guide RNA architecture (A) Schematic of the sgRNA and target DNA. The disordered regions are enclosed by dashed boxes. PK, pseudoknot; R:AR, crRNA repeat–tracrRNA anti-repeat duplex. (B) Structure of the sgRNA and target DNA complex.

The Cas12c2 sgRNA (nucleotides A[−93]–U17) consists of the 17-nucleotide guide segment (nucleotides G1–U17) and the 93-nucleotide RNA scaffold (nucleotides A[−93]–G[−1]), in which the 18-nucleotide crRNA-derived sequence and the 71-nucleotide tracrRNA-derived sequence are connected by a GAAA tetraloop (Figures 2A and 2B). The present structure revealed that the Cas12c2 RNA scaffold comprises four stem regions (stems 1–4), two pseudoknot regions (PK 1 and PK 2), and a linker region (Linker), although the apical regions of stem 3 (nucleotides [−44]–[−30]) and stem 4 (nucleotides [−29]–[−12]) were not included in the model due to the ambiguous density. Notably, the RNA scaffold of Cas12c2 is distinct from those previously predicted from its nucleotide sequence (Harrington et al., 2020; Yan et al., 2019) (Figures 2A and 2B). Unlike the tracrRNAs for Cas9 and some Cas12 enzymes, which extensively base pair with their crRNAs, the tracrRNAs for the Cas12c family enzymes contain short (3–5 nucleotide) sequences (docks) complementary to their crRNA repeat sequences (anchors) and were thus referred to as a short-complementarity untranslated RNA (scoutRNA) rather than a tracrRNA (Harrington et al., 2020). In the present structure, nucleotides G(−4)–G(−1) in the crRNA-derived anchor sequence base pair with nucleotides C(−55)–C(−58) in the tracrRNA-derived sequence (corresponding to the scoutRNA dock sequence) to form PK 2 (crRNA repeat–tracrRNA anti-repeat duplex 2 [R:AR-2]). Contrary to the prediction, nucleotides U(−11)–G(−8) in the crRNA also base pair with nucleotides A(−88)–A(−91) in the tracrRNA (scoutRNA) to form PK 1 (R:AR-1). In addition, nucleotides U(−18)–U(−12) in the crRNA probably base pair with nucleotides A(−23)–A(−29) in the tracrRNA (scoutRNA) to form stem 4 (R:AR-3), although it is disordered in the present structure. Given that the Cas12c2 tracrRNA extensively base pairs with the crRNA repeat region, it should be referred to as a tracrRNA rather than a scoutRNA. As predicted from the nucleotide sequence, C(−87)–U(−78) and G(−45)–A(−54) form an RNA duplex (stem 1). Nucleotides G(−74)–C(−62) and C(−44)–G(−30) form stem 2 and stem 3, respectively, while the peripheral nucleotides of stem 2 (A[−69]–U[−67]) and stem 3 (G[−41]–A[−33]) are not resolved in the density map. Nucleotides U(−77)–U(−75) and G(−61)–A(−59) in the Linker region adopt single-stranded conformations and connect stem 2 with stem 1 and PK 2, respectively. Stem 1 and PK 1 coaxially stack with PK 2/stem 3 and stem 4, respectively, to form a unique X-junction structure (Figure 2A). Taken together, the present structure has revealed that the crRNA and tracrRNA of Cas12c2 form the guide RNA scaffold through extensive base pairing.

### RNA scaffold recognition

**Figure 3:**
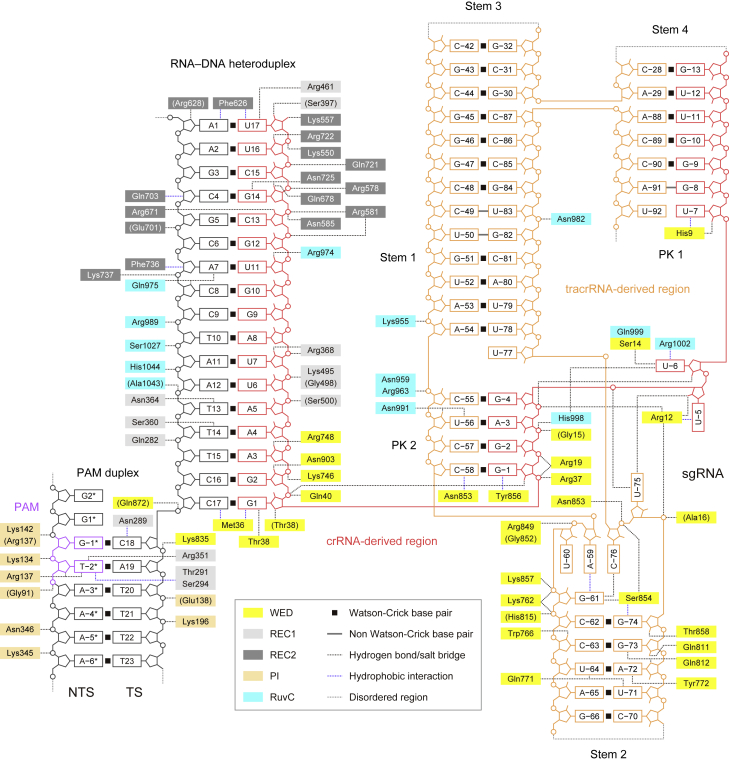
Schematic of nucleic-acid recognition The residues that interact with the nucleic acids through their main chains are shown in parentheses. The disordered regions are indicated by dashed gray lines.

**Figure 4:**
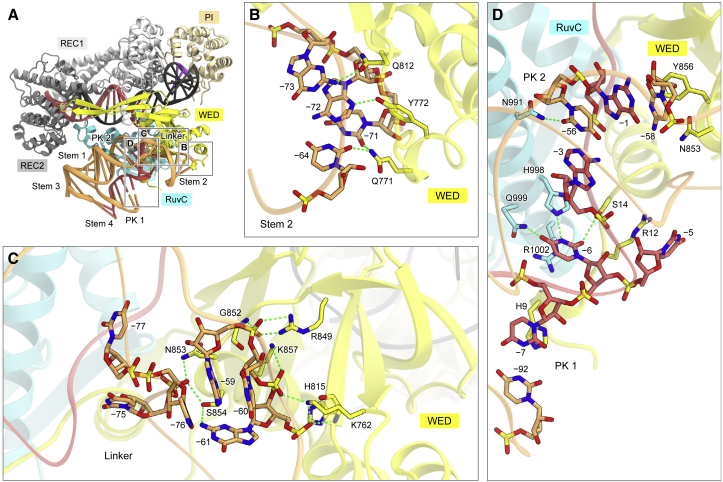
Guide RNA recognition (A) Recognition sites of the guide RNA scaffold. (B–D) Recognition of stem 2 (B), Linker (C), and PK 1–PK 2 (D).

In the Cas12c2 structure, the guide RNA scaffold is recognized by the WED and RuvC domains (Figures 3 and 4A). Stem 1 is recognized by the helix α1 and the α1–α2 linker in the RuvC domain through sugar-phosphate backbone interactions (Figure 3). The backbone phosphate groups of G(−82), A(−54), and A(−53) interact with Asn982, Asn959/Arg963, and Lys955, respectively. Stem 2 and the Linker are recognized by the WED domain (Figures 4B and 4C). G(−73), A(−72), and U(−71)/U(−64) form hydrogen bonds with Gln812, Tyr772, and Gln771, respectively (Figure 4B). Linker is mainly recognized by Cas12c2 through sugar-phosphate backbone interactions (Figure 4C). The backbone phosphate groups of U(−75), G(−61), U(−60), and A(−59) interact with Asn853/Ser854, Lys762/His815, Lys762/Lys857, and Arg849/Gly852, respectively. In addition, G(−61) forms a base-specific hydrogen bond with Ser854. A(−59) stacks with C(−76) and U(−60), stabilizing the Linker conformation. PK 1 has fewer interactions with the protein, and only U(−7) stacks with His9 in the WED domain (Figure 4D). PK 2 is recognized by the RuvC domain through sugar-phosphate backbone interactions with the crRNA-derived region. The ribose and nucleobase moieties of U(−56) form hydrogen bonds with Asn991, while the last C(−58)-G(−1) base pair in PK 2 stacks with Asn853 and Tyr856 (Figure 4D). The nucleobase of U(−6) in PK 1–PK 2 forms hydrogen bonds with Ser14, His998, and Gln999. Moreover, the nucleobases of U(−6) and U(−5) stack with Arg1002 and Arg12, respectively. Stem 3 and stem 4 are mostly disordered in the present structure, and do not interact with the protein. Altogether, the present structure has revealed the mechanism of the guide RNA recognition by Cas12c2.

### Target DNA recognition

**Figure 5:**
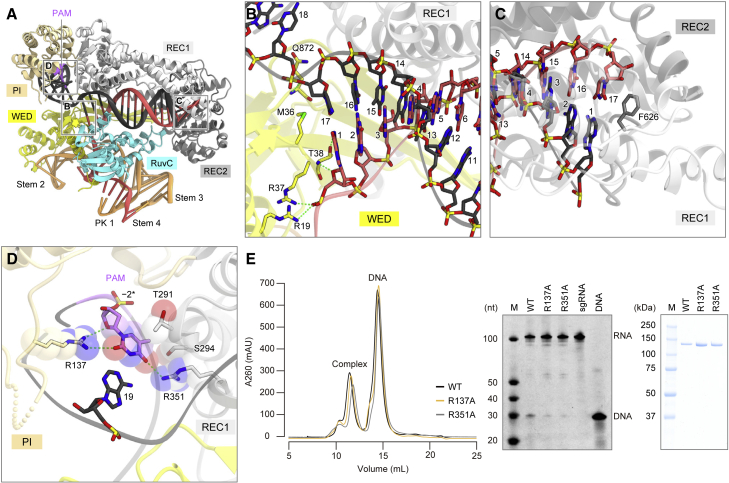
Target DNA recognition (A) Recognition sites of the target DNA. (B–D) Recognition of the guide RNA–target DNA heteroduplex (B and C) and the PAM duplex (D). (E) DNA-binding activities of the WT Cas12c2 and the Cas12c2 mutants. The peak fractions containing the complex were analyzed by SDS-PAGE and urea-PAGE.

The guide RNA–target DNA heteroduplex is accommodated within the positively charged central channel formed by the REC1 and RuvC domains and recognized by Cas12c2 through interactions with its sugar-phosphate backbone (Figures 3 and 5A). Met36 in the WED domain stacks with the first G1-dC17 base pair in the heteroduplex, while Arg19/Arg37 and Gln872 interact with the backbone phosphate groups of G1 and dC18, respectively (Figure 5B), thereby facilitating the heteroduplex formation. Phe626 in the REC2 domain stacks with the U17-dA1 base pair in the heteroduplex, as observed in Cas12a (Yamano et al., 2016) (Figure 5C), indicating that 17 nucleotides in the spacer sequence function as a guide segment in Cas12c2-mediated DNA recognition. The displaced single-stranded NTS in the target dsDNA is not visible in the present structure, in contrast to some in the other Cas12 structures, such as Cas12a (Swarts and Jinek, 2019) and Cas12e (Liu et al., 2019), in which the displaced NTS binds to the positively charged groove formed by the RuvC and TNB domains. These differences suggested that the NTS in the R-loop interacts with the RuvC domain of Cas12c2 less stably, as compared to those in the other Cas12 enzymes. Together, these structural observations explain the mechanism of the RNA-guided DNA targeting by Cas12c2.

### PAM recognition

The PAM duplex is recognized between the WED, PI, and REC1 domains (Figures 5A and 5D). The nucleotide dT(−2^∗^) in the TG PAM extensively interacts with the PI and REC domains, whereas the other nucleotides in the PAM duplex do not form base-specific contacts with the protein. Specifically, Arg137 in the PI domain and Arg351 in the REC1 domain form hydrogen bonds with the O4′/O2 and O6 atoms of dT(−2^∗^), respectively (Figure 5D). In addition, the 5-methyl group of dT(−2^∗^) forms van der Waals interactions with Thr291 and Ser294 in the REC1 domain. Indeed, the R137A and R351A mutations abolished the DNA-binding activities of Cas12c2 (Figure 5E), confirming the functional importance of Arg137 and Arg351 for the PAM recognition. These results explain the mechanism of the short TN PAM recognition by Cas12c2.

### Cryo-EM structure of the Cas12c2–guide RNA binary complex

**Figure 6:**
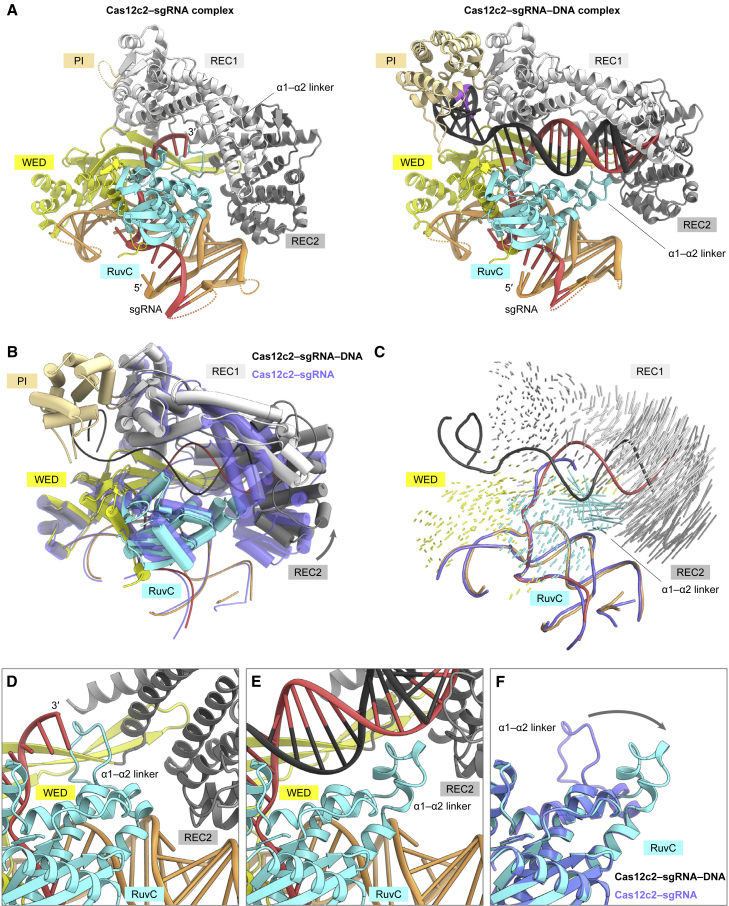
Structural comparison between the Cas12c2 binary and ternary complexes (A) Overall structures of the Cas12c2–sgRNA binary complex (left) and the Cas12c2–sgRNA–target DNA ternary complex (right). The disordered regions are indicated as dotted lines. (B) Superimposition of the ternary complex (colored as in A) and the binary complex (light blue). (C) Structural comparison between the binary and ternary complexes. Vector length correlates with the domain transition scale. The structural image was prepared using PyMOL (http://www.pymol.org). (D and E) Structures of the α1–α2 linker region in the binary complex (D) and the ternary complex (E). (F) Superimposition of the RuvC domains in the ternary complex (cyan) and the binary complex (light blue). See also Figures S4 and S5.

To elucidate how Cas12c2 assembles with the sgRNA prior to the target DNA binding, we determined the cryo-EM structure of Cas12c2 in complex with the sgRNA at an overall resolution of 3.0 Å (Figures 6A and S4A–S4D; Table 1). A structural comparison between the binary and ternary complexes revealed structural changes in the REC, PI, and RuvC domains upon target DNA binding (Figures 6B and 6C). The Cas12c2–sgRNA–target DNA ternary complex adopts an open conformation, in which the REC lobe is distant from the RuvC domain and forms the central channel that accommodates the RNA–DNA heteroduplex. In contrast, the Cas12c2–sgRNA binary complex adopts a closed conformation, in which the REC2 domain interacts with stem 3 of the tracrRNA. Notably, the PI domain is disordered in the binary complex structure, suggesting that the PI domain of Cas12c2 is flexible and stabilized by the interaction with the PAM duplex, whereas that of Cas12a is pre-ordered prior to DNA binding (Yamano et al., 2017). A structural comparison also revealed a local conformational change in the α1–α2 linker in the RuvC domain (Figures 6D–6F). In the ternary complex, the α1–α2 linker contains short α helices and interacts with the REC2 domain and the RNA–DNA heteroduplex (Figure 6E). In the binary complex, the α1–α2 linker adopts a loop conformation and interacts with the REC2 and WED domains (Figure 6D), thereby stabilizing the closed conformation. The binary complex also revealed that only the first six nucleotides of the guide segment are ordered in the central channel (Figure 6A), suggesting that nucleotides 1–6 in the guide segment serve as the important seed region for the R-loop formation, as in Cas12a (Swarts et al., 2017) and Cas12b (Yang et al., 2016). The α1–α2 linker is located close to the ordered terminal nucleotide (U6), implying that the linker region facilitates the bending of the guide segment in the binary complex.

In Cas12b and Cas12i, a short α-helix between the strand β4 and the helix α3 in the RuvC domain (referred to as the Lid motif) undergoes conformational changes upon the target DNA binding and interacts with the guide–target heteroduplex, thereby opening the RuvC active site for the substrate DNA binding (Figures S5A and S5B) (Zhang et al., 2020). In contrast, the corresponding region (residues 1014–1022) of Cas12c2 adopts similar loop conformations between the binary and ternary complexes (Figure S5C), suggesting that the Lid motif is not involved in the RuvC domain activation in Cas12c2, as in Cas12a (Figure S5D) (Swarts et al., 2017).

### Pre-crRNA processing mechanism

**Figure 7:**
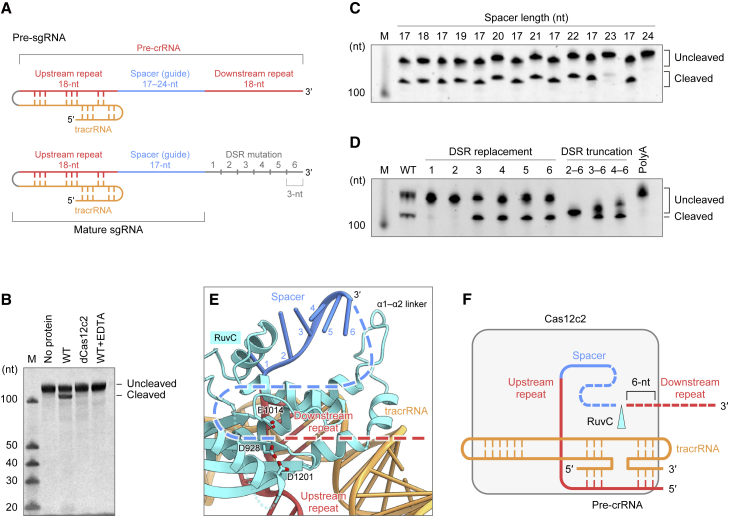
Pre-crRNA processing (A) Schematic of the pre-sgRNA used for the processing experiments. (B) *In vitro* pre-sgRNA processing activities of the WT Cas12c2, dCas12c2 (D928A), and WT Cas12c2 in the presence of EDTA. The cleavage products were analyzed by 10% denaturing urea-PAGE. (C and D) *In vitro* pre-sgRNA processing activities of the WT Cas12c2 for the pre-sgRNAs with different spacer lengths (17–24-nucleotides) (C) and those with different downstream repeat sequences (D). The cleavage products were analyzed by 8% (C) or 10% (D) denaturing urea-PAGE. (E) RuvC active site in the Cas12c2 binary complex. The catalytic residues are shown as stick models. The possible trajectory of the bound pre-sgRNA is shown as a dashed line. (F) Proposed model of the pre-crRNA processing by Cas12c2. See also Figure S6.

A recent study showed that Cas12c processes its pre-crRNA through a unique ruler mechanism, in which Cas12c recognizes the repeat sequence upstream of the spacer sequence (upstream repeat) and processes the 3′ end of the pre-crRNA at 18-nucleotides downstream of the recognized upstream repeat sequence (Harrington et al., 2020). To confirm the Cas12c2-catalyzed pre-crRNA processing, we added the 18-nucleotide downstream repeat sequence after the 3′ end of the sgRNA to prepare a pre-sgRNA (Figure 7A) and performed *in vitro* processing experiments. Like the pre-crRNA–tracrRNA hybrid used in a previous study (Harrington et al., 2020), the pre-sgRNA was processed by Cas12c2 (Figure 7B). To confirm the ruler mechanism, we examined the processing of pre-sgRNAs with a 17–24-nucleotide spacer sequence and found that Cas12c2 efficiently processes the pre-sgRNAs with the 17–22-nucleotide spacers, but not those with the 23–24-nucleotide spacers (Figure 7C). Notably, the lengths of the cleaved sgRNAs increased according to their spacer lengths (Figure 7C), indicating that Cas12c2 recognizes the downstream repeat of pre-crRNAs and cleaves the pre-crRNA between the spacer and downstream repeat. To test this hypothesis, we measured the processing activity of Cas12c2 for a pre-sgRNA-polyA, in which the downstream repeat was replaced with an 18-nucleotide polyA sequence. Cas12c2 failed to process the pre-sgRNA-polyA (Figure 7D), indicating that Cas12c2 recognizes not only the upstream repeat sequence, but also the downstream repeat sequence for the pre-crRNA processing. These results are inconsistent with the proposed ruler mechanism, in which Cas12c2 recognizes the upstream repeat sequence, but not the downstream repeat sequence (Harrington et al., 2020). Furthermore, we examined the effects of mutations in the downstream repeat for the pre-crRNA processing. First, we investigated whether Cas12c2 processes six pre-sgRNA replacement mutants (DSR-replacements 1–6), in which the 18-nucleotide downstream repeat was divided into six segments (segments 1–6) and three nucleotides in each of segments 1–6 were replaced with their complementary nucleotides, respectively (Figure 7A). Cas12c2 cleaved the DSR-replacements 3–6, but not the DSR-replacements 1 and 2 (Figure 7D). Next, we examined whether Cas12c2 processes three pre-sgRNA truncation mutants (DSR-truncations 2–6, 3–6, and 4–6), in which the segments 2–6, 3–6, and 4–6 were truncated, respectively (Figure 7A). Cas12c2 cleaved the DSR-truncations 3–6 and 4–6, but not the DSR-truncation 2–6 (Figure 7D). Together, these results indicated that the first six nucleotides of the downstream repeat are important for the Cas12c2-mediated pre-crRNA processing.

While Cas12a and Cas12i process their pre-crRNAs at their WED domains (Swarts et al., 2017; Zhang et al., 2020; Huang et al., 2020; Zhang et al., 2021) and Cas12j process its pre-crRNA at its RuvC domain (Pausch et al., 2020), the catalytic site for the Cas12c2-mediated pre-crRNA processing remains unknown. We examined the processing activities of wild-type (WT) Cas12c2 and dCas12c2 (D928A) and found that dCas12c2 cannot process the pre-sgRNA (Figures 7B and 7E). In addition, WT Cas12c2 lacked processing activity in the presence of EDTA (Figure 7B), indicating a metal-dependent mechanism. These results suggested that Cas12c2 processes the pre-sgRNA at the RuvC active site in a metal-dependent manner, as in Cas12j (Pausch et al., 2020). Taken together, our biochemical data indicated that Cas12c2 processes its pre-crRNA through a unique mechanism, in which Cas12c2 recognizes both the upstream and downstream repeat sequences and cleaves the pre-crRNA immediately upstream of the downstream repeat sequence in the RuvC active site (Figure 7F).

## Discussion

In this study, we determined the cryo-EM structures of the Cas12c2–sgRNA binary and Cas12c2–sgRNA–target DNA ternary complexes. The structures revealed that, while Cas12c2 has the conserved WED and RuvC domains, the REC and PI domains of Cas12c2 are structurally distinct from those of the other Cas12 enzymes. In addition, the structures demonstrated that the guide RNA scaffold of Cas12c2 has a unique X-junction architecture, which was not predicted from its nucleotide sequence and differs from those of the other Cas12 enzymes. Thus, our findings highlighted the structural diversity in the type V CRISPR-Cas effector proteins.

Our biochemical data indicated that, unlike other Cas12 enzymes, Cas12c2 recognizes the first six nucleotides of the downstream repeat region of the pre-crRNA in the presence of the tracrRNA and processes the 3′ end of the pre-crRNA using the RuvC active site (Figures 7F and S6A–S6D). In the Cas12c2–sgRNA complex structure, the first six nucleotides within the 17-nucleotide spacer sequence are ordered, and the α1–α2 linker in the RuvC domain is located near the ordered sixth spacer nucleotide (Figure 7E), suggesting that the linker region facilitates the bending of the pre-crRNA toward the RuvC active site for the processing reaction. In the dCas12c2–pre-sgRNA complex structure, which we also determined, the first six nucleotides of the spacer are ordered—as in the Cas12c2–sgRNA complex structure—while the rest of the spacer and the downstream repeat are disordered (data not shown). These structural observations suggested that the downstream repeat region of the pre-crRNA transiently binds to the RuvC and TNB domains during the processing reaction. Cas12c2 did not cleave the pre-sgRNAs with the 23–24-nucleotide spacers, suggesting that the long spacers inhibit the recruitment of the downstream repeat region to the RuvC active site of Cas12c2.

Cas12c2 and Cas12j (Pausch et al., 2020) process their pre-crRNAs at their RuvC domains. Our comparisons of the binary complex structures of Cas12c2 and Cas12j (Pausch et al., 2021) revealed some structural differences between them (Figures S6A and S6B). In the Cas12c2 structure, the sixth nucleotide in the 17-nucleotide spacer sequence is blocked by the α1–α2 linker in the RuvC domain, and the rest of the spacer is disordered (Figure S6A), suggesting that the pre-crRNA is kinked at this position and extends toward the RuvC active site for the pre-crRNA processing. Unlike Cas12c2, Cas12j has a longer Lid motif in the RuvC domain and the REC2 domain is inserted between the α1 and α2 helices in the RuvC domain (Figure S6B). In the Cas12j structure, the 13 nucleotides in the spacer region are ordered and interact with the Lid motif and the REC2 domain. While Cas12c and Cas12j process the 3′ and 5′ ends of their pre-crRNAs, respectively, the 5′ end of the mature crRNA is located away from the RuvC active site in the Cas12j binary complex structure (Figure S6B). In addition, Cas12c2 and Cas12j share the RuvC active sites formed by the conserved catalytic residues (Asp928, Glu1014, and Asp1201 in Cas12c2). Thus, the structures of Cas12c2 and Cas12j in complex with their pre-crRNAs will be needed to elucidate their pre-crRNA processing mechanisms. Unlike Cas12c2 and Cas12j, Cas12a and Cas12i process their pre-crRNAs through metal-independent, acid-base catalytic mechanisms, in which His or Lys residues in the WED domain serve as acid-base catalyst pairs (Swarts et al., 2017; Zhang et al., 2020; Huang et al., 2020; Zhang et al., 2021) (Figures S6C and S6D). Together, these findings highlighted the mechanistic diversity of the pre-crRNA processing by the Cas12 family enzymes.

Our biochemical data indicated that Cas12c2 lacks dsDNA cleavage activity *in vitro*, consistent with a previous study (Harrington et al., 2020), although Cas12c2 has a similar RuvC active site to those of the other Cas12 enzymes. Notably, the present structure revealed that the TNB domain of Cas12c2 is flexible and disordered, whereas those of the other Cas12 enzymes interact with their RuvC domains and facilitate target DNA loading into the RuvC active site. These structural differences suggested that Cas12c2 lacks DNA cleavage activity, possibly due to its flexible TNB domain. A previous study showed that Cas12c2, but not dCas12c2, mediates dsDNA interference in bacterial cells (Yan et al., 2019), suggesting that the RuvC domain is involved in the Cas12c2-mediated interference, although it does not catalyze dsDNA cleavage. It is possible that Cas12c2 binds dsDNA targets and suppresses their transcription, as recently reported in the type II systems (Ratner et al., 2019; Workman et al., 2021), and that dCas12c2 failed to mediate dsDNA interference due to the absence of the RuvC-mediated pre-crRNA processing. Our biochemical data also revealed that, unlike Cas12c2 and OspCas12c, Cas12c1 cleaves dsDNA substrates. While the catalytic residues in their RuvC domains are highly conserved, their RuvC and TNB domains share limited sequence identity (20%–30%), suggesting that the observed differences in their DNase activities are due to the structural variations in these domains. Thus, the Cas12c1 structure would provide clues toward the elucidation of the functional differences of the Cas12c proteins.

The present structure revealed the TN PAM recognition mechanism of Cas12c2. Previous studies demonstrated that the Cas12 enzymes recognize diverse PAM sequences in distinct manners. For example, Cas12a recognizes a TTTV PAM by using the PI domain inserted within the WED domain (Yamano et al., 2016; Swarts and Jinek, 2019). In contrast, Cas12c2 has the PI domain inserted within the REC domain, and recognizes the T nucleobase in the PAM, using Arg137 in the PI domain and Arg351 in the REC domain. The PI domain of Cas12c2 is structurally unrelated to those of the other Cas12 enzymes. These observations highlighted the mechanistic diversity of the PAM recognition by the Cas12 family enzymes.

The PAM requirement limits the target space in CRISPR-mediated genome engineering. To expand the target space, previous studies identified a variety of natural Cas enzymes with different PAM specificities (Ran et al., 2015; Zetsche et al., 2015), and engineered Cas variants with altered PAM recognition have also been developed (Kleinstiver et al., 2015; Nishimasu et al., 2018; Walton et al., 2020). For example, the Cas9 variants, SpCas9-NG and SpG, efficiently recognize an NG sequence as the PAM (Nishimasu et al., 2018; Walton et al., 2020). Notably, Cas12c2 is presently the only known natural Cas enzyme that recognizes a single nucleotide as the PAM. Thus, although Cas12c2 lacks DNA cleavage activity, it could be used as an RNA-guided DNA targeting platform with broad target ranges in genome-engineering applications, such as gene modulation and epigenome editing. In summary, our findings enhance the understanding of diverse type V CRISPR-Cas12 effectors and provide the molecular framework for the development of efficient genome-engineering technologies.

### Limitations of the study

Our structural and functional analyses provided mechanistic insights into the unique pre-crRNA processing and PAM recognition by Cas12c. However, it is unclear how Cas12c2 recognizes the downstream repeat sequence and processes its pre-crRNAs using the RuvC domain. It is also unknown why, unlike Cas12c1, Cas12c2 lacks the target DNA cleavage activity. Therefore, further studies will be required to fully understand the action mechanisms of the Cas12c family enzymes. Furthermore, it will be important to examine whether Cas12c2 can be used for gene regulation in mammalian cells.

## STAR★Methods

### Key resources table

**Table:** 

REAGENT or RESOURCE	SOURCE	IDENTIFIER
**Chemicals, peptides, and recombinant proteins**		

Cas12c (Cas12c1, Cas12c2, and OspCas12c)	Yan et al., 2019	N/A
Cas12c2, various mutants	This paper	N/A
SpCas9 D10A	Nishimasu et al., 2018	N/A
AsCas12a	Nishimasu et al., 2018	N/A

**Deposited data**		

Cas12c2 binary complex coordinates	This paper	PDB: 7V93
Cas12c2 ternary complex coordinates	This paper	PDB: 7V94
Cas12c2 binary complex EM map	This paper	EMDB: EMD-31807
Cas12c2 ternary complex EM map	This paper	EMDB: EMD-31808

**Experimental models: Cell lines**		

*E. coli* Mach1	Thermo Fisher Scientific	C862003
*E. coli* Rosetta 2 (DE3)	Novagen	71397

**Oligonucleotides**		

DNA primers	This paper	Table S1
DNA oligos (for structure determination)	This paper	Table S1
Cas12c guide RNAs	Yan et al., 2019	Table S1
Cas12c pre-sgRNA, and various mutants	This paper	Table S1

**Recombinant DNA**		

pET28a-mH6-Cas12c1	This paper	https://benchling.com/s/seq-iW2bxdrClXAO2yegbugM?m=slm-6IRAra8rGu1N7GwReQfu
pET28a-mH6-Cas12c2	This paper	https://benchling.com/s/seq-47rEdAQC7K8KoN91Lkx9?m=slm-gCp3vxVeR31tK5cv04u3
pET28a-mH6-OspCas12c	This paper	https://benchling.com/s/seq-3fGZqpsX5xKWy82T0pZR?m=slm-1MXDGa5XzJI8qgEXngq0
pET28a-mH6-Cas12c2, various mutants	This paper	https://benchling.com/s/seq-47rEdAQC7K8KoN91Lkx9?m=slm-gCp3vxVeR31tK5cv04u3
pUC119-T20	This paper	https://benchling.com/s/seq-i4shHg70wpvZqBBDHO7z?m=slm-UwFbajmRIvpMlQlL4phb
pET-SpCas9 D10A	Nishimasu et al., 2018	N/A
pET-AsCas12a	Nishimasu et al., 2018	N/A

**Software and algorithms**		

SerialEM	Mastronarde, 2005	https://bio3d.colorado.edu/SerialEM/
MotionCor2	Zheng et al., 2017	https://emcore.ucsf.edu/ucsf-software
Relion	Zivanov et al., 2018	https://www3.mrc-lmb.cam.ac.uk/relion/index.php?title=Main_Page
CTFFIND4	Rohou and Grigorieff, 2015	https://grigoriefflab.umassmed.edu/ctffind4
Servalcat	Yamashita et al., 2021	https://github.com/keitaroyam/servalcat
COOT	Emsley et al., 2010; Nicholls et al., 2018	https://www2.mrc-lmb.cam.ac.uk/personal/pemsley/coot/
PHENIX	Afonine et al., 2018	https://www.phenix-online.org/
MolProbity	Chen et al., 2010	https://www.phenix-online.org/documentation/reference/molprobity_tool.html
UCSF-Chimera	Pettersen et al., 2004	https://www.rbvi.ucsf.edu/chimera
CueMol	N/A	http://www.cuemol.org
Larch Python library	Newville, 2013	https://xraypy.github.io/xraylarch/

**Other**		

Amicon Ultra-4 Centrifugal Filter Units - 10,000 NMWL	Millipore	UFC801024
Ni-NTA Superflow	QIAGEN	30450
HiTrap SP HP	GE Healthcare	17115201
Superdex 200 Increase 10/300	GE Healthcare	28990944
HiLoad 16/600 Superdex 200	GE Healthcare	28989335
300 mesh R 1.2/1.3 holey carbon Au	Quantifoil	https://www.quantifoil.com/products/quantifoil/quantifoil-circular-holes/
300 mesh R 1.2/1.3 holey carbon Cu/Rh	Quantifoil	https://www.quantifoil.com/products/quantifoil/quantifoil-circular-holes/

### Resource availability

#### Lead contact

Further information and requests for resources and reagents should be directed to and will be fulfilled by the lead contact, Osamu Nureki (nureki@bs.s.u-tokyo.ac.jp).

#### Material availability

All unique/stable reagents generated in this study are available from the lead contact with a completed Materials Transfer Agreement.

### Experimental model and subject details

*E. coli* cells were cultured at 37°C in LB medium (containing 20 mg/l kanamycin) for plasmid and protein preparation.

### Method details

#### Protein and RNA preparation

The His_6_-tagged Cas12c proteins (Cas12c1, Cas12c2, and OspCas12c) were expressed in *Escherichia coli* Rosetta2 (DE3) (Novagen), using the pET28a-mH6-Cas12c vectors (Addgene plasmids #120872 and #120873) (Yan et al., 2019). The *E. coli* Rosetta2 (DE3) cells were cultured at 37°C in LB medium (containing 20 mg/l kanamycin) until the OD_600_ reached 0.8, and the protein expression was then induced by the addition of 0.1 mM isopropyl β-D-thiogalactopyranoside (Nacalai Tesque). The *E. coli* cells were further cultured at 20°C overnight, and harvested by centrifugation. The *E. coli* cells were resuspended in buffer A (50 mM Tris-HCl, pH 8.0, 20 mM imidazole, and 1 M NaCl), lysed by sonication, and then centrifuged. The supernatant was mixed with 3 mL Ni-NTA Superflow resin (QIAGEN), and the mixture was loaded into an Econo Column (Bio-Rad). The protein was eluted with buffer B (20 mM Tris-HCl, pH 8.0, 0.3 M imidazole, and 0.3 M NaCl). The protein was then loaded onto a 5 mL HiTrap SP HP column (GE Healthcare), equilibrated with buffer C (20 mM Tris-HCl, pH 8.0, and 0.3 M NaCl). The protein was eluted with a linear gradient of 0.3–2 M NaCl. The protein was further purified by chromatography on a HiLoad 16/600 Superdex 200 column (GE Healthcare), equilibrated in buffer D (20 mM Tris-HCl, pH 8.0, and 0.5 M NaCl). The purified protein was stored at −80°C until use. The mutations were introduced by a PCR-based method, and the sequences were confirmed by DNA sequencing. The sgRNAs were transcribed *in vitro* with T7 RNA polymerase, and purified by 8% denaturing (7 M urea) polyacrylamide gel electrophoresis.

#### Electron microscopy sample preparation

The Cas12c2–sgRNA–target DNA ternary complex was reconstituted by mixing the purified Cas12c2, the 112-nucleotide sgRNA (110 nucleotides plus 5′ GG for *in vitro* transcription), the 33-nucleotide target DNA strand (Sigma-Aldrich), and the 33-nucleotide non-target DNA strand (Sigma-Aldrich), at a molar ratio of 1:1.2:2:2 (Table S1). The Cas12c2–sgRNA binary complex was reconstituted by mixing the purified Cas12c2 and the sgRNA at a molar ratio of 1:1.2. The ternary and binary complexes were purified by size-exclusion chromatography on a Superdex 200 Increase 10/300 column (GE Healthcare), equilibrated with buffer E (10 mM Tris-HCl, pH 8.0, 150 mM NaCl, and 2 mM MgCl_2_). The ternary and binary complex samples (3 μl, ∼1.5 mg/mL) were applied to a freshly glow-discharged Au 300 mesh R1.2/1.3 grid (Quantifoil) and a Cu/Rh 300 mesh R1.2/1.3 grid (Quantifoil), respectively, in a Vitrobot Mark IV (FEI) at 4°C with a blotting time of 4 s under 100% humidity conditions. The grids were plunge-frozen in liquid ethane cooled at liquid nitrogen temperature.

#### Electron microscopy data collection and processing

The cryo-EM data were collected using a Titan Krios G3i microscope (Thermo Fisher Scientific), running at 300 kV and equipped with a Gatan Quantum-LS Energy Filter (GIF) and a Gatan K3 Summit direct electron detector, operated in the electron counting mode (ternary complex) or the electron counting CDS mode (binary complex). Each video was recorded at a nominal magnification of 105,000 × , corresponding to a calibrated pixel size of 0.83 Å. The electron flux for the ternary complex was set to 13 e^−^/pix/sec for 2.6 s, resulting in accumulated exposures of 50 e^−^/Å^2^. The electron flux for the binary complex was set to 7.5 e^−^/pix/sec for 5.0 s, resulting in accumulated exposures of 54 e^–^/Å^2^. The data were automatically acquired by the image shift method using the SerialEM software (Mastronarde, 2005), with a defocus range of −0.8 to −1.6 μm, and about 3,000 videos were obtained. The dose-fractionated videos were subjected to beam-induced motion correction and dose-weighting, using the MotionCor2 algorithm (Zheng et al., 2017) implemented in RELION-3 (Zivanov et al., 2018), and the contrast transfer function (CTF) parameters were estimated using CTFFIND4 (Rohou and Grigorieff, 2015).

The ternary complex data were processed using RELION-3.0. From 2,925 motion-corrected and dose-weighted micrographs, 1,465,975 particles were initially picked using a 2D reference, and extracted at a pixel size of 3.19 Å. These particles were subjected to several rounds of 2D and 3D classifications. The selected 534,196 particles were then re-extracted at a pixel size of 1.08 Å and subjected to 3D refinement, CTF refinement, and Bayesian polishing (Zivanov et al., 2019). These particles were then processed by 3D refinement, and subsequent postprocessing of the map improved its global resolution to 2.7 Å, according to the Fourier shell correlation (FSC) = 0.143 criterion (Rosenthal and Henderson, 2003). The local resolution was estimated by RELION-3.1.

The binary complex data were processed using RELION-3.1. From 3,267 motion-corrected and dose-weighted micrographs, 2,852,137 particles were initially picked using a 2D reference, and extracted at a pixel size of 3.32 Å. These particles were subjected to several rounds of 2D and 3D classifications. The selected 289,394 particles were then re-extracted at a pixel size of 1.10 Å and subjected to 3D refinement, Bayesian polishing, 3D refinement with SIDESPLITTER (Ramlaul et al., 2020), and CTF refinement. These particles were subjected to 3D refinement again, and subsequent postprocessing of the map improved its global resolution to 3.0 Å, according to the FSC = 0.143 criterion. The local resolution was estimated by RELION-3.1.

#### Model building and validation

The initial model of the ternary complex was automatically built using the Buccaneer pipeline (Hoh et al., 2020) from the CCP-EM package (Burnley et al., 2017). The manual model rebuilding was performed using COOT (Emsley et al., 2010; Nicholls et al., 2018), with the aid of a density map calculated by deepEMhancer (Sanchez-Garcia et al., 2021). The model of the binary complex was built using the model of the ternary complex. The models were refined using phenix.real_space_refine ver. 1.16 (Afonine et al., 2018) and REFMAC5 (Nicholls et al., 2018) in the Servalcat pipeline (Yamashita et al., 2021), with the secondary structure and base pair/stacking restraints. The structure validation was performed using MolProbity (Chen et al., 2010). In the model of the ternary complex, residues 1–4, 141–144, 558–566, 913–918, 1081–1198, and 1218 of Cas12c2, nucleotides (−93), (−69)–(−67), (−41)–(−33), and (−27)–(−14) of the sgRNA, nucleotides (−8)–(−1) and 24–25 of the TS, and nucleotides (−8^∗^)–(−7^∗^) and 3^∗^–25^∗^ of the NTS are not included in the final model, since these regions are not well resolved in the density map. In the model of the binary complex, residues 1–4, 91–238, 558–566, 626–630, 913–918, 1081–1198, and 1218 of Cas12c2, and nucleotides (−93), (−69)–(−67), (−41)–(−33), (−27)–(−14) and 7–17 of the sgRNA are not included in the final model. The FSC curves representing model versus full map were calculated using Servalcat, based on the final model and the full, unfiltered, and unsharpened map. The statistics of the 3D reconstruction and model refinement are summarized in Table 1. Molecular graphics figures were prepared with UCSF Chimera (Pettersen et al., 2004), PyMOL (http://www.pymol.org), and CueMol (http://www.cuemol.org).

#### X-ray fluorescence analysis

X-ray fluorescence spectra were collected on BL41XU at SPring-8 (Hyogo, Japan), using an XR-100 FAST SDD detector (Amptek) and a digital pulse processor PX5 (Amptek). The frozen samples were irradiated with an 18.00-keV X-ray beam, and X-ray fluorescence spectra were accumulated for 180 s and 205 s from the Cas12c2 protein sample and a blank (buffer D), respectively. X-ray emission line data were obtained from the Larch Python library (Newville, 2013).

#### Size-exclusion chromatography

The DNA-binding activities of the Cas12c2–sgRNA complex were evaluated by size-exclusion chromatography. The purified Cas12c2 protein (WT, R137A, or R351A), the sgRNA, and the target dsDNA were mixed at a 1:1.2:2 molar ratio in buffer E (400 μl), and the mixture was then analyzed using a Superdex 200 Increase 10/300 column, equilibrated with buffer E.

#### *In vitro* DNA cleavage experiments

The Cas12c–guide RNA complex (1.25 μM) was prepared by mixing each purified Cas12c protein (Cas12c1, Cas12c2 and OspCas12c) (2.5 μM) with its cognate guide RNAs (crRNA and tracrRNA) (5 μM each) at 37°C for 5 min, in 5 μL buffer E. Each pre-assembled complex (2 μl, 1.25 μM, 250 nM final concentrations) was mixed with circular and linearized plasmid targets containing the 18-nucleotide target sequence and the TTTG PAM (8 μl, 100 ng), and then incubated at 37°C in a 10 μL reaction mixture (5 mM Tris-HCl, pH 7.5, 100 mM KCl, 5 mM MgCl_2_, and 1 mM DTT) for 30 min. The reaction was stopped by the addition of Proteinase K (1 μl, 6.7 ng), and the products were resolved on a 0.8% agarose gel and then visualized with Midori Green Xtra (Nippon Genetics). *Acidaminococcus sp.* Cas12a (AsCas12a) and the *Streptococcus pyogenes* Cas9 (SpCas9) D10A mutant were prepared as described (Nishimasu et al., 2018), and the purified AsCas12a (TTTG PAM) and SpCas9 D10A (AGG PAM) were used as dsDNase and nickase controls, respectively. *In vitro* cleavage experiments were performed at least three times.

#### *In vitro* processing experiments

The pre-sgRNAs were prepared by adding the 18-nucleotide downstream repeat sequence after the 3′ end of the 17–24-nucleotide spacer sequence of the sgRNA. The pre-sgRNA-polyA was constructed by adding the 18-nucleotide polyA sequence after the 3′ end of the 17-nucleotide spacer sequence of the sgRNA. The sgRNA replacement mutants (DSR-replacements 1–6) were generated by replacing three nucleotides in segments 1–6 in the 18-nucleotide downstream repeat with their complementary nucleotides, respectively. The pre-sgRNA truncation mutants (DSR-truncations 2–6, 3–6, and 4–6) were prepared by truncating the segments 2–6, 3–6, and 4–6 in the 18-nucleotide downstream repeat, respectively. The purified Cas12c2 protein (WT or dCas12c2) and the pre-sgRNAs were mixed at a molar ratio of 1:1, in buffer E with or without 0.5 mM EDTA, and then incubated at 37°C for 60 min. The reactions were stopped by the addition of Proteinase K (13 ng), and then analyzed by urea-PAGE. The gels were stained with SYBR Gold (Invitrogen). *In vitro* processing experiments were performed at least three times.

### Quantification and statistical analysis

*In vitro* experiments were performed at least three times. Data are shown as mean ± SD (n = 3).

## Data Availability

• The structural models and density maps have been deposited in the Protein Data Bank under the accession codes PDB: 7V93 (binary complex) and PDB: 7V94 (ternary complex). The raw images have been deposited in the Electron Microscopy Public Image Archive under the accession codes EMDB: EMD-31807 (binary complex) and EMDB: EMD-31808 (ternary complex). The data of unprocessed image files have been deposited in the Mendeley Data repository (https://doi.org/10.17632/hshkzhjy3x.1). • This paper does not report original code. • Any additional information required to reanalyze the data reported in this paper is available from the lead contact upon request.

## Supplemental information

Document S1. Figures S1–S6 and Table S1

Document S2. Article plus supplemental information
