# Supplementary material for: Structure of the type V-C CRISPR-Cas effector enzyme
Source: Mol Cell. 2022 May 19;82(10):1865–1877.e4. doi: 10.1016/j.molcel.2022.03.006 (PMC9522604; doi:10.1016/j.molcel.2022.03.006)
Supplement: Document S1. Figures S1–S6 and Table S1 [file mmc1.pdf]

Molecular Cell, Volume *82*

Supplemental information

Structure of the type V-C

CRISPR-Cas effector enzyme

Nina Kurihara, Ryoya Nakagawa, Hisato Hirano, Sae Okazaki, Atsuhiro Tomita, Kan Kobayashi, Tsukasa Kusakizako, Tomohiro Nishizawa, Keitaro Yamashita, David A. Scott, Hiroshi Nishimasu, and Osamu Nureki

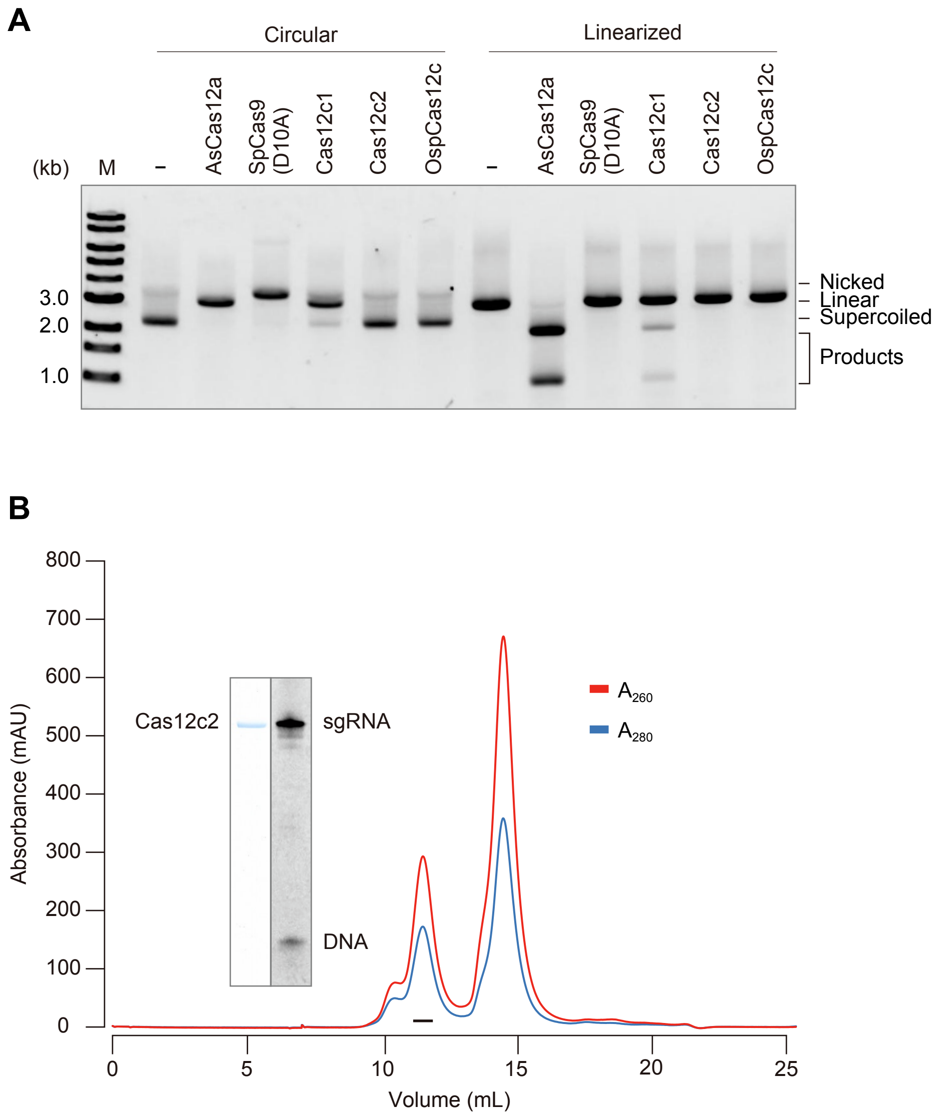

Figure S1. Biochemical characterization, Related to Figure 1

(A) *In vitro* dsDNA cleavage experiments. The circular and linearized plasmid targets with the TTTG PAM were incubated with Cas protein–guide RNA complexes at 37°C for 30 min, and the cleavage products were then analyzed by fractionation on a 0.8% agarose gel. AsCas12a (TTTG PAM) and SpCas9 (D10A) (AGG PAM) were used as dsDNase and nickase controls, respectively.

(B) Size-exclusion chromatography profile of the Cas12c2–sgRNA–target DNA complex. The peak fraction (indicated by a black bar) was analyzed by SDS-PAGE and urea-PAGE.

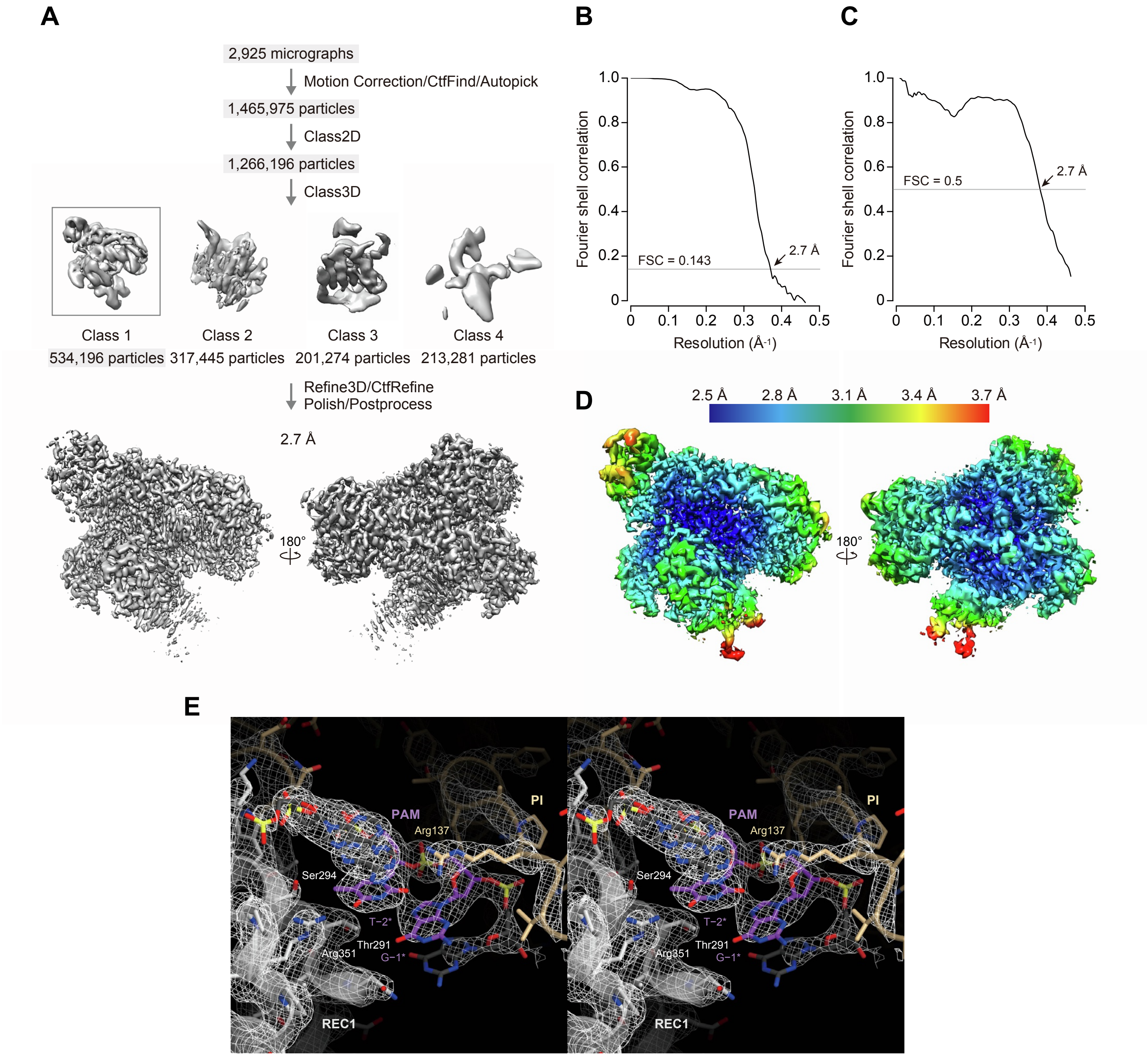

Figure S2. Cryo-EM analysis of the ternary complex, Related to Figure 1

(A) Single-particle cryo-EM image processing workflow.

(B) Fourier shell correlation curve calculated between the half-maps in the 3D reconstruction.

(C) Fourier shell correlation curve calculated between the refined model and the density map.

(D) Local resolution of the density map.

(E) Density map (unsharpened, FSC-weighted) for the PAM recognition site (stereo view).

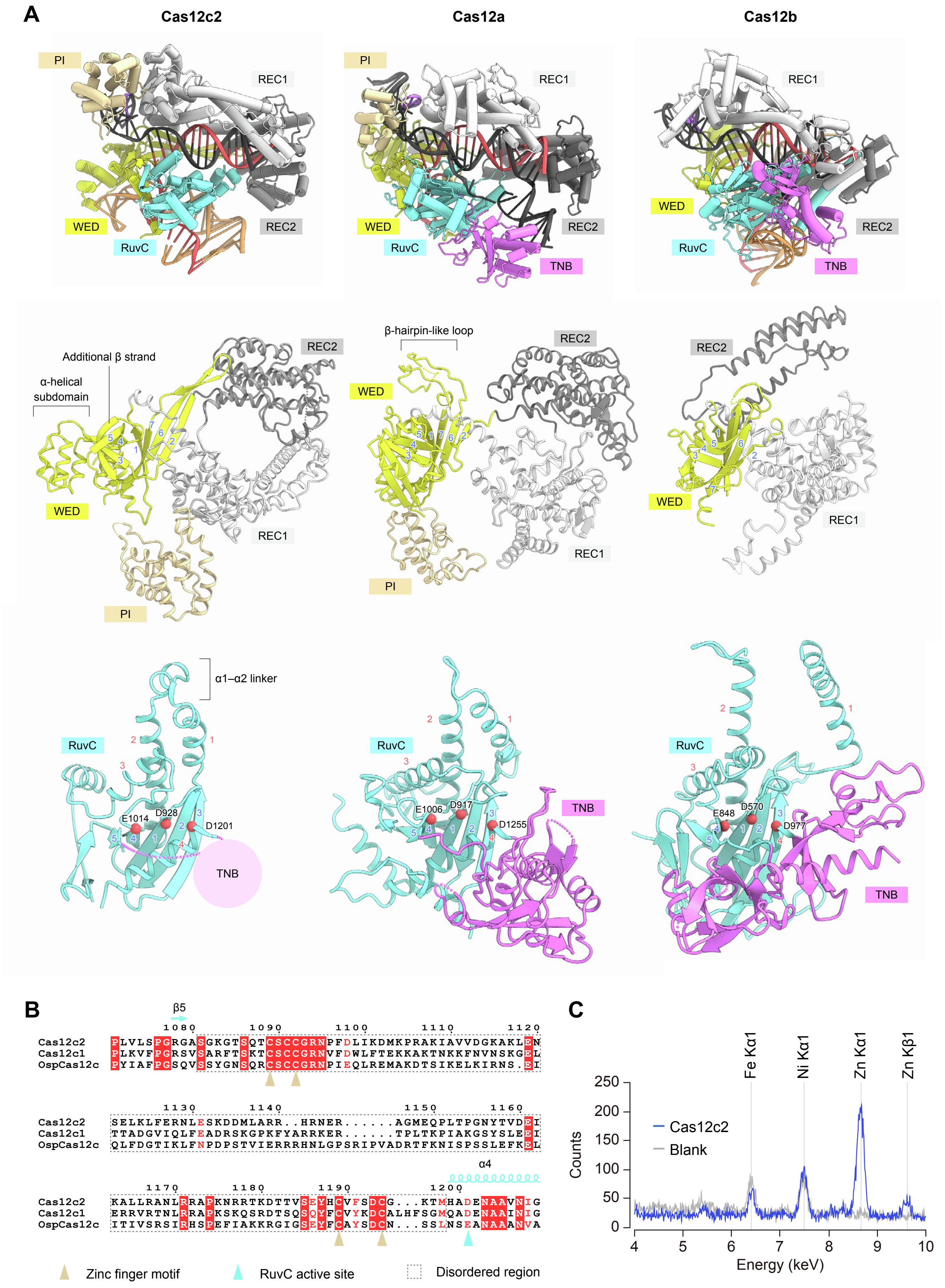
A
Cas12c2
Cas12a
Cas12b
PI
REC1
WED
RuvC
REC2
TNB
Additional β strand
α-helical subdomain
β-hairpin-like loop
α1–α2 linker
E1014
D928
D1201
E1006
D917
D1255
E848
D570
D977
B
β5
1080
1090
1100
1110
1120
Cas12c2 PLVLSPGRGASGKGTSQTCSCCGRNPFDLIKDMKPRAKIAVVDGKAKLEN
Cas12c1 PLKVFPGRSVSARFTSKTCSCCGRNVFDWLFTEKKAKTNKKFNVNSKGEL
OspCas12c PYIAFPGSQVSSYGNSQRCSCCGRNPIEQLREMAKDTSIKELKIRNS.EI
1130
1140
1150
1160
Cas12c2 SELKLFERNLESKDDMLARR..HRNER......AGMEQPLTPGNYTVDEI
Cas12c1 TTADGVIQLFEADRSKGPKFYARRKER......TPLTKPIAKGSYSLEEI
OspCas12c QLFDGTIKLFNPDPSTVIERRRHNLGPSRIPVADRTFKNISPSSLEFKEL
α4
1170
1180
1190
1200
Cas12c2 KALLRANLRRAPKNRRTKDTTVSEYHCVFSDCG...KTMHADENAAVNIG
Cas12c1 ERRVRTNLRRAPKSKQSRDTSQSQYFCVYKDCALHFSGMQADENAAINIG
OspCas12c ITIVSRSIRHSPEFIAKKRGIGSEYFCAYSDCN...SSLNSEANAAANVA
Zinc finger motif
RuvC active site
Disordered region
C
Fe Kα1
Ni Kα1
Zn Kα1
Zn Kβ1
Counts
Energy (keV)
Cas12c2
Blank
0
50
100
150
200
250
4
5
6
7
8
9
10

Figure S3. Domain structures, Related to Figure 1

(A) Structural comparison of Cas12c2 with Cas12a from *Francisella novicida* (PDB ID: 6I1K) (Swarts and Jinek, 2019) and Cas12b from *Alicyclobacillus acidoterrestris* (PDB ID: 5U33) (Yang et al., 2016). The Cas12 enzymes commonly adopt a bilobed architecture containing the structurally similar WED and RuvC domains, despite their limited sequence identity (the conserved α helices (red) and β strands (blue) are numbered). The WED and RuvC domains comprise an OB fold and an RNase H fold, respectively. The PI domain of Cas12a is inserted within the WED domain, whereas that of Cas12c2 is inserted within the REC1 domain. The predicted location of the TNB domain of Cas12c2 is indicated by a magenta circle. The TNB domains are inserted between the conserved strand β5 and helix α4 in the RuvC domains.

(B) Sequence comparison of the TNB domains of Cas12c2, Cas12c1, and OspCas12c. The secondary structure of Cas12c2 is indicated above the sequences. The key residues are marked by triangles. The disordered regions, which correspond to the TNB domains, are enclosed in a dashed box. The TNB domain of Cas12c2 contains two zinc-finger motifs (CXXC and CXXXXC). The figure was prepared using Clustal Omega (http://www.ebi.ac.uk/Tools/msa/clustalo) and ESPript3 (http://espript.ibcp.fr/ESPript/ESPript).

(C) X-ray fluorescence analysis. X-ray fluorescence spectra were collected from the purified Cas12c2 and the sample buffer (blank). Zn Kα and Kβ signals were only detected from the protein sample. Fe and Ni signals are derived from the beamline optics.

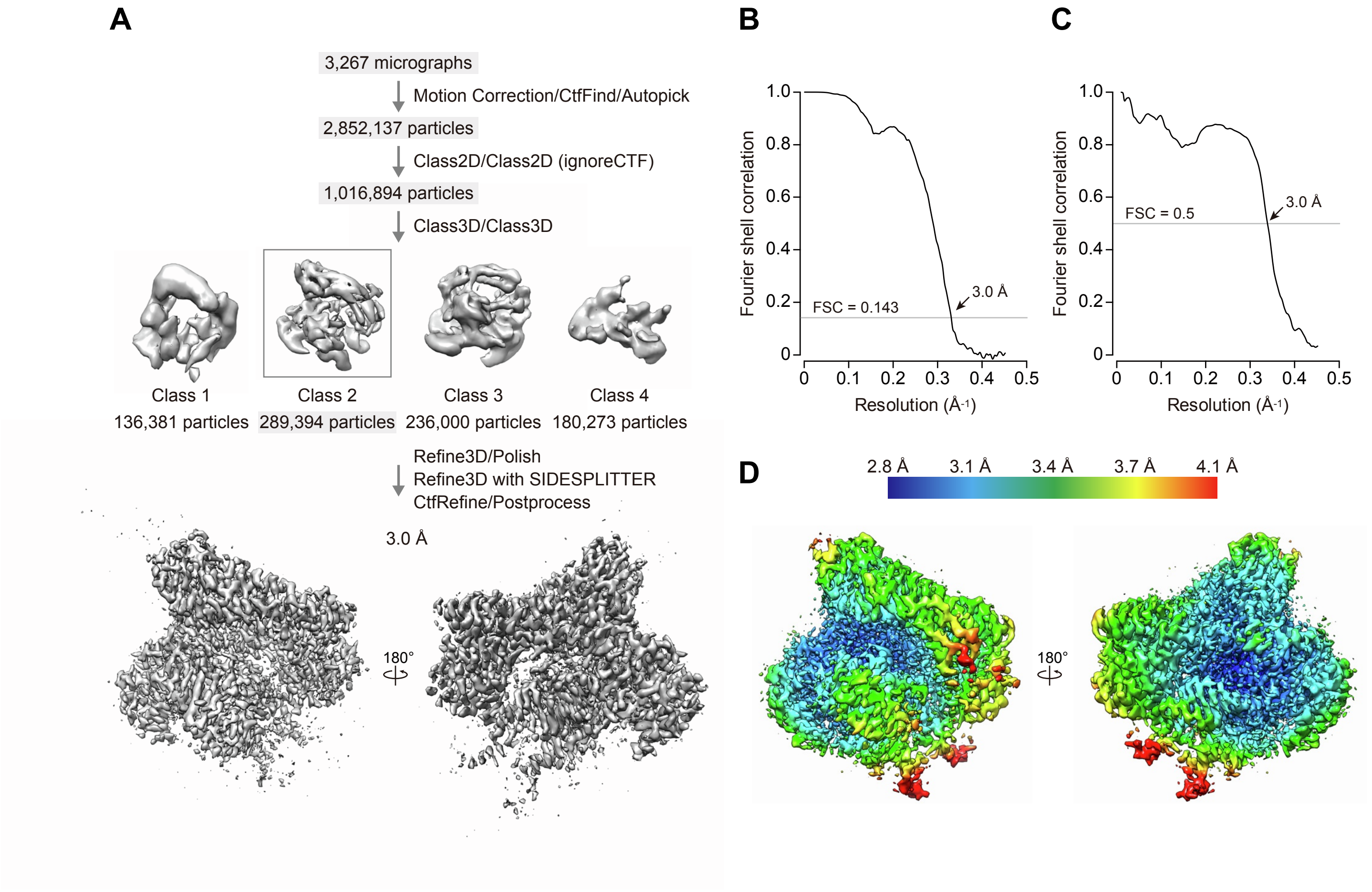

Figure S4. Cryo-EM analysis of the binary complex, Related to Figure 6

(A) Single-particle cryo-EM image processing workflow.

(B) Fourier shell correlation curve calculated between the half-maps in the 3D reconstruction.

(C) Fourier shell correlation curve calculated between the refined model and the density map.

(D) Local resolution of the density map.

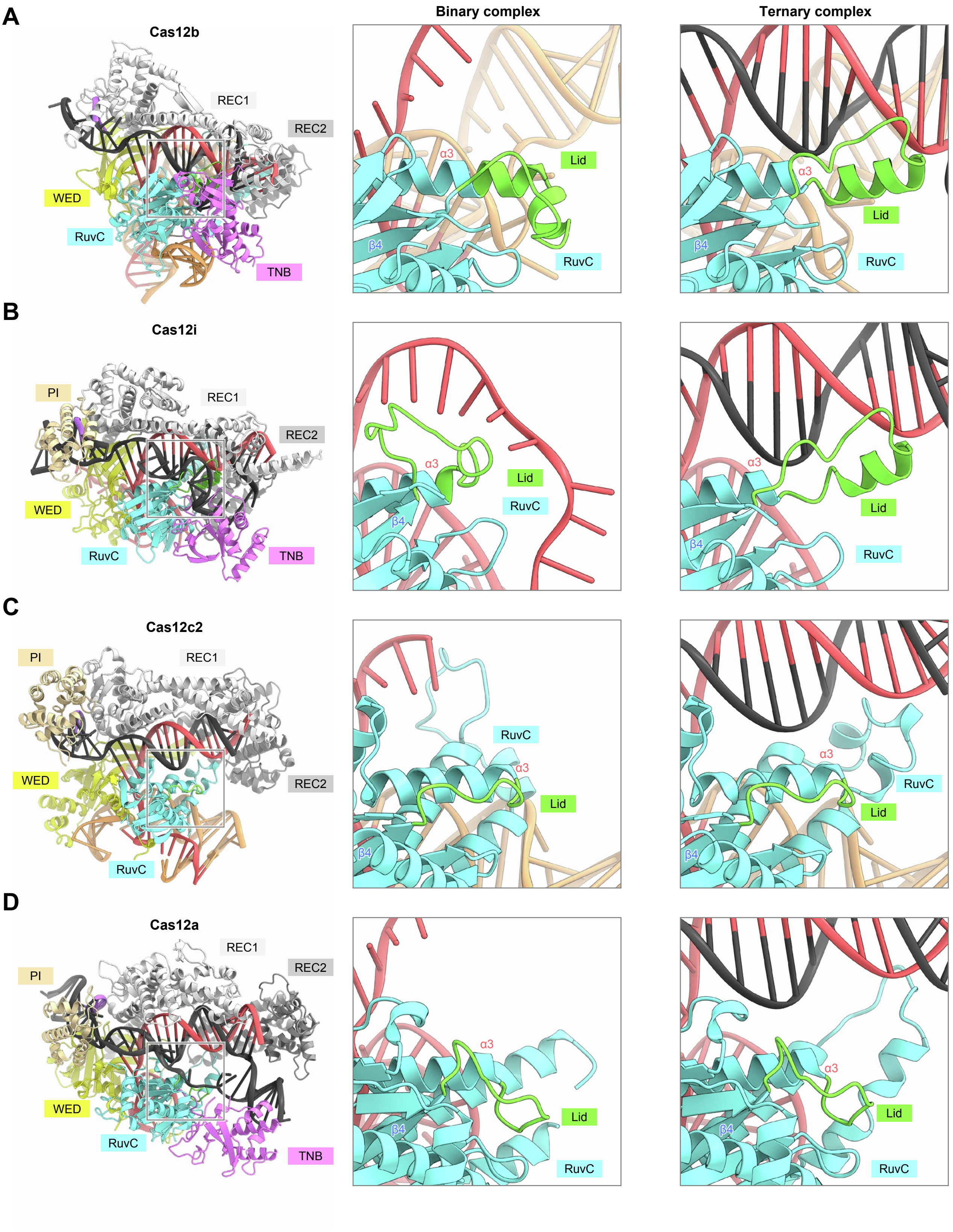

Figure S5. Lid motifs of the Cas12 family enzymes, Related to Figure 6

(A–D) Overall structures of the ternary complexes and close-up views of the Lid motifs in the binary and ternary complexes of Cas12b from *Alicyclobacillus acidoterrestris* (PDB IDs: 5U33 and 5U34) (Yang et al., 2016) (A), Cas12i1 (PDB IDs: 7D8C and 7D2L) (Zhang et al., 2021) (B), Cas12c2 (C), and Cas12a from *F. novicida* (PDB IDs: 5NG6 and 6I1K) (Swarts et al., 2017; Swarts and Jinek, 2019) (D).

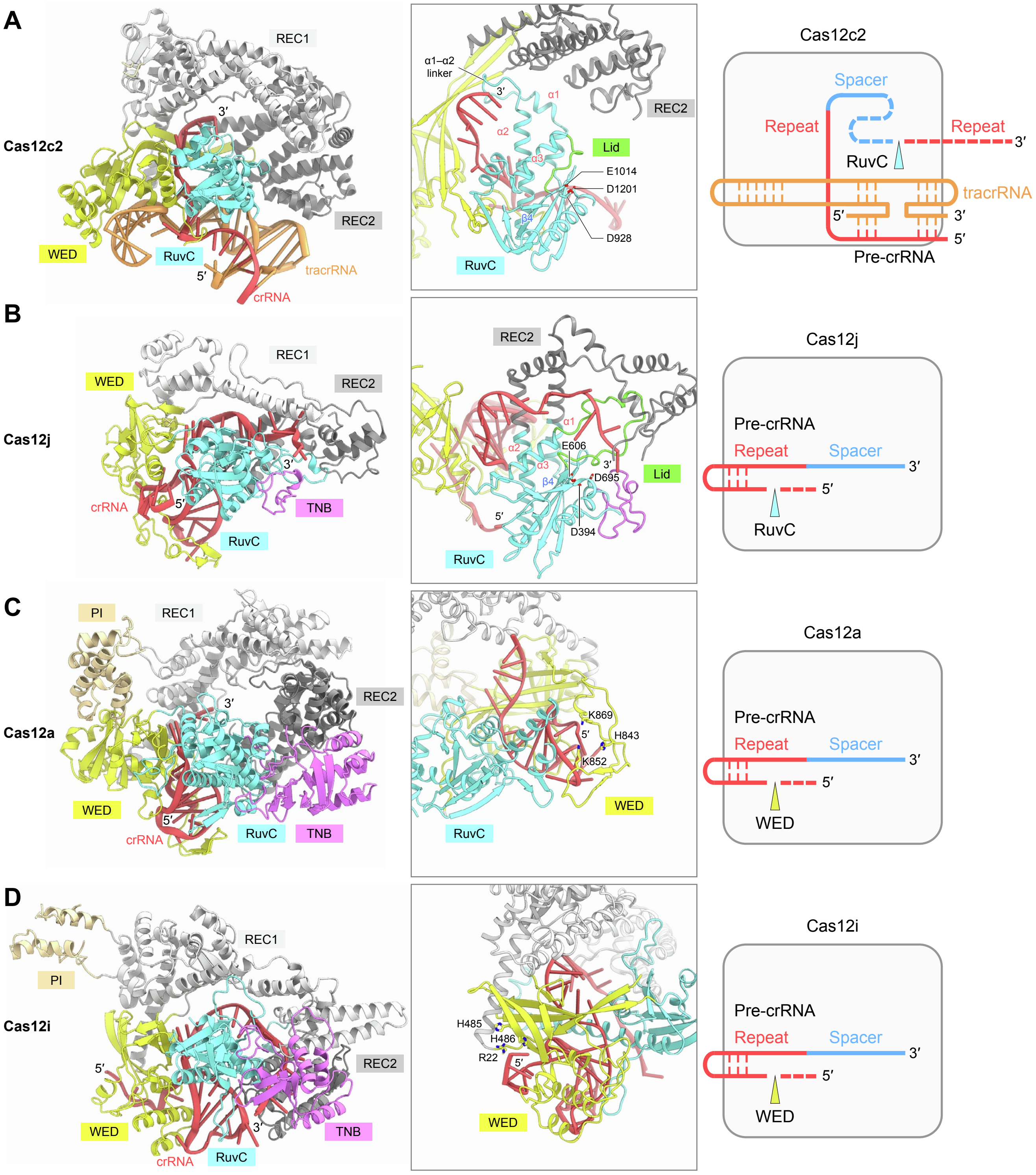

Figure S6. Pre-crRNA processing mechanisms of the Cas12 family enzymes, Related to Figure 7
(A–D) Overall structures of the binary complexes, catalytic sites for pre-crRNA processing, and schematics of pre-crRNA processing of Cas12c2 (A), Cas12j2 (PDB ID: 7M5O) (Pausch et al., 2021) (B), Cas12a from *F. novicida* (PDB ID: 5NG6) (Swarts et al., 2017) (C), and Cas12i1 (PDB ID: 7D8C) (Zhang et al., 2021) (D). REC1/tracrRNA and REC1 are omitted for clarity in the middle panels of (A) and (B), respectively.

Table S1. Nucleic-acid sequences used in this study, Related to STAR Methods

Oligonucleotides used to introduce the Cas12c2 mutations (pET28a-His$_6$-Cas12c2)		
Mutation	Forward primer	Reverse primer
D928A	gcgCTGGGTGAACGTGGCCTGGG	AATCGCAACAAAACGATCGAACAGCATC
R137A	gcgGAGGTTGCGAAGAAAGATGGTAGCAAG	CGGCGCTTTGGTGAAACGTTCG
R351A	GTTCGGtgcGAACGGCGCGTAGTTTTTCTTGC	CCGTTCgcaCCGAACTTTGGTGGCAAGATCGAT
The guide RNA and target DNA used for the structure determination		
sgRNA	ggAUACCACCCGUGCAUUUCUGGAUCAAUGAUCCGUACCUCAAUGUCCGGGCGCGCAGCUAGAGCGACCUGAAGAAAUUCAGGUUGGGUUUGAGGGGAAAUUAGGUGCGCUU	
Target DNA strand	AGGTTGCCAAGCGCACCTAATTTCCCATTTTAG	
Non-target DNA strand	CTAAAATGGGAAATTAGGTGCGCTTGGCAACCT	
Pre-sgRNAs used for the processing experiments		
Pre-sgRNA-polyA	ggAUACCACCCGUGCAUUUCUGGAUCAAUGAUCCGUACCUCAAUGUCCGGGCGCGCAGCUAGAGCGACCUGAAGAAAUUCAGGUUGGGUUUGAGGGGAAAUUAGGUGCGCUUAAAAAAAAAAAAAAAAAA	
Pre-sgRNA-17	ggAUACCACCCGUGCAUUUCUGGAUCAAUGAUCCGUACCUCAAUGUCCGGGCGCGCAGCUAGAGCGACCUGAAGAAAUUCAGGUUGGGUUUGAGGGGAAAUUAGGUGCGCUUUUCAGGUUGGGUUUGAGG	
Pre-sgRNA-18	ggAUACCACCCGUGCAUUUCUGGAUCAAUGAUCCGUACCUCAAUGUCCGGGCGCGCAGCUAGAGCGACCUGAAGAAAUUCAGGUUGGGUUUGAGGGGAAAUUAGGUGCGCUUGUUCAGGUUGGGUUUGAGG	
Pre-sgRNA-19	ggAUACCACCCGUGCAUUUCUGGAUCAAUGAUCCGUACCUCAAUGUCCGGGCGCGCAGCUAGAGCGACCUGAAGAAAUUCAGGUUGGGUUUGAGGGGAAAUUAGGUGCGCUUGGUUCAGGUUGGGUUUGAGG	
Pre-sgRNA-20	ggAUACCACCCGUGCAUUUCUGGAUCAAUGAUCCGUACCUCAAUGUCCGGGCGCGCAGCUAGAGCGACCUGAAGAAAUUCAGGUUGGGUUUGAGGGGAAAUUAGGUGCGCUUGGCUUCAGGUUGGGUUUGAGG	
Pre-sgRNA-21	ggAUACCACCCGUGCAUUUCUGGAUCAAUGAUCCGUACCUCAAUGUCCGGGCGCGCAGCUAGAGCGACCUGAAGAAAUUCAGGUUGGGUUUGAGGGGAAAUUAGGUGCGCUUGGCAUUCAGGUUGGGUUUGAGG	
Pre-sgRNA-22	ggAUACCACCCGUGCAUUUCUGGAUCAAUGAUCCGUACCUCAAUGUCCGGGCGCGCAGCUAGAGCGACCUGAAGAAAUUCAGGUUGGGUUUGAGGGGAAAUUAGGUGCGCUUGGCAAUUCAGGUUGGGUUUGAGG	
Pre-sgRNA-23	ggAUACCACCCGUGCAUUUCUGGAUCAAUGAUCCGUACCUCAAUGUCCGGGCGCGCAGCUAGAGCGACCUGAAGAAAUUCAGGUUGGGUUUGAGGGGAAAUUAGGUGCGCUUGGCAACUUCAGGUUGGGUUUGAGG	
Pre-sgRNA-24	ggAUACCACCCGUGCAUUUCUGGAUCAAUGAUCCGUACCUCAAUGUCCGGGCGCGCAGCUAGAGCGACCUGAAGAAAUUCAGGUUGGGUUUGAGGGGAAAUUAGGUGCGCUUGGCAACCUUCAGGUUGGGUUUGAGG	
DSR-replacement 1	ggAUACCACCCGUGCAUUUCUGGAUCAAUGAUCCGUACCUCAAUGUCCGGGCGCGCAGCUAGAGCGACCUGAAGAAAUUCAGGUUGGGUUUGAGGGGAAAUUAGGUGCGCUUAAGAGGUUGGGUUUGAGG	
DSR-replacement 2	ggAUACCACCCGUGCAUUUCUGGAUCAAUGAUCCGUACCUCAAUGUCCGGGCGCGCAGCUAGAGCGACCUGAAGAAAUUCAGGUUGGGUUUGAGGGGAAAUUAGGUGCGCUUUUCUCCUUGGGUUUGAGG	
DSR-replacement 3	ggAUACCACCCGUGCAUUUCUGGAUCAAUGAUCCGUACCUCAAUGUCCGGGCGCGCAGCUAGAGCGACCUGAAGAAAUUCAGGUUGGGUUUGAGGGGAAAUUAGGUGCGCUUUUCAGGAACGGUUUGAGG	
DSR-replacement 4	ggAUACCACCCGUGCAUUUCUGGAUCAAUGAUCCGUACCUCAAUGUCCGGGCGCGCAGCUAGAGCGACCUGAAGAAAUUCAGGUUGGGUUUGAGGGGAAAUUAGGUGCGCUUUUCAGGUUGCCAUUGAGG	
DSR-replacement 5	ggAUACCACCCGUGCAUUUCUGGAUCAAUGAUCCGUACCUCAAUGUCCGGGCGCGCAGCUAGAGCGACCUGAAGAAAUUCAGGUUGGGUUUGAGGGGAAAUUAGGUGCGCUUUUCAGGUUGGGUAACAGG	
DSR-replacement 6	ggAUACCACCCGUGCAUUUCUGGAUCAAUGAUCCGUACCUCAAUGUCCGGGCGCGCAGCUAGAGCGACCUGAAGAAAUUCAGGUUGGGUUUGAGGGGAAAUUAGGUGCGCUUUUCAGGUUGGGUUUGUCC	
DSR-truncation 2–6	ggAUACCACCCGUGCAUUUCUGGAUCAAUGAUCCGUACCUCAAUGUCCGGGCGCGCAGCUAGAGCGACCUGAAGAAAUUCAGGUUGGGUUUGAGGGGAAAUUAGGUGCGCUUUUC	
DSR-truncation 3–6	ggAUACCACCCGUGCAUUUCUGGAUCAAUGAUCCGUACCUCAAUGUCCGGGCGCGCAGCUAGAGCGACCUGAAGAAAUUCAGGUUGGGUUUGAGGGGAAAUUAGGUGCGCUUUUCAGG	
DSR-truncation 4–6	ggAUACCACCCGUGCAUUUCUGGAUCAAUGAUCCGUACCUCAAUGUCCGGGCGCGCAGCUAGAGCGACCUGAAGAAAUUCAGGUUGGGUUUGAGGGGAAAUUAGGUGCGCUUUUCAGGUUG	

The codons for the mutations and the 5′ GG for *in vitro* transcription are indicated with lower case letters. The guide/target and tetraloop sequences are underlined.
